# Glymphatic dysfunction as a biomarker for post-stroke cognitive impairment

**DOI:** 10.1038/s41598-025-04054-9

**Published:** 2025-06-03

**Authors:** Sheng Zhang, Weitao Yu, Xiaofan Zhang, Haoyue Cheng, Zheyu Zhang, Shouxuan Gao, Jie Zheng, Liang Yu, Peng Wang, Yu Geng, Jie Zhang, Faliang Gao

**Affiliations:** 1 Center for Rehabilitation Medicine, Department of Neurology, Zhejiang Provincial People’s Hospital (Affiliated People’s Hospital), Hangzhou Medical College, Hangzhou, 310016 Zhejiang China; 2 The Second School of Clinical Medicine, Hangzhou Normal University, Hangzhou, 310006 Zhejiang China; 3 Department of Neurology, Tongji Hospital, Huazhong University of Science and Technology, Wuhan, 430030 China; 4 Department of Epidemiology & Health Statistics, School of Public Health, School of Medicine, Zhejiang University, Hangzhou, 310058 China; 5 Department of Neurology, The Second Affiliated Hospital of Zhejiang University, School of Medicine, Hangzhou, 310009 China; 6 Department of Radiology, Zhejiang Provincial People’s Hospital (Affiliated People’s Hospital), Hangzhou Medical College, Hangzhou, 310016 Zhejiang China; 7 Center for Rehabilitation Medicine, Department of Rehabilitation, Zhejiang Provincial People’s Hospital (Affiliated People’s Hospital), Hangzhou Medical College, Hangzhou, 310016 Zhejiang China; 8 Center for Rehabilitation Medicine, Department of Neurosurgery, Zhejiang Provincial People’s Hospital (Affiliated People’s Hospital), Hangzhou Medical College, Hangzhou, 310016 Zhejiang China; 9 Center for Rehabilitation Medicine, Department of Neurosurgery, Zhejiang Provincial People’s Hospital, Affiliated People’s Hospital, Hangzhou Medical College, Hangzhou, 310014 Zhejiang China; 10 Center for Rehabilitation Medicine, Department of Rehabilitation, Zhejiang Provincial People’s Hospital, Affiliated People’s Hospital, Hangzhou Medical College, Hangzhou, 310014 Zhejiang China

**Keywords:** Stroke, Glymphatic clearance function, DTI-ALPS index, Prognosis, Poststroke cognitive impairment, Diffusion tensor imaging, Biomarkers, Diseases, Neurology

## Abstract

Ischemic stroke impacts glymphatic function, but its role in prognosis remains unclear. This study evaluated glymphatic function in 146 participants, including non-stroke (healthy controls, n = 48; nonvascular cognitive impairment patients, n = 47) and ischemic stroke cohorts (n = 51). The bilateral diffusion tensor imaging analysis along the perivascular space (DTI-ALPS) index, choroid plexus (CP), and perivascular space (PVS) volume ratio, which represent the glymphatic system, were compared across two cohorts and between pre-rehabilitation (Time 1) and 30 days post-rehabilitation (Time 2). Post-stroke cognitive impairment (PSCI) was characterized as enduring cognitive deficits persisting six months after a stroke. Stroke patients exhibited significantly lower bilateral DTI-ALPS index compared with the non-stroke population (*P* < 0.05), while the infarct side showed post-rehabilitation improvement (*P* < 0.05). The DTI-ALPS index of infarct side at Time 1 did not predict poor outcome but was correlated with 6-month PSCI (*P* < 0.05). These results indicate that ischemic stroke diminishes glymphatic function, partially recovering post-rehabilitation, and suggest that the DTI-ALPS index could serve as a predictor for cognitive impairment following ischemic stroke.

## Introduction

Stroke is the second leading cause of death globally, with ischemic stroke (IS) comprising the vast majority^1^. In China, IS, accounting for about 69.6% of stroke cases^2^. Due to the high prevalence of morbidity and mortality after stroke, numerous studies primarily focused on therapies at ultra-early stage, such as thrombolysis, thrombectomy, and neuroprotective therapy. Despite efforts, still about 50% patients encounter reperfusion injury^3^, and face the significant challenge of unreversible motor dysfunction and potential cognitive decline.

The glymphatic system, a recently identified mechanism responsible for clearing waste from the nervous system, offers valuable insights into stroke recovery. This system operates by facilitating the exchange of cerebrospinal fluid (CSF) and interstitial fluid (ISF) through perivascular pathways, such as para-arterial influx channels, para-venous efflux channels, and astrocyte-mediated connections^4^. Previous studies have shown that glymphatic system dysfunction exacerbates brain edema in the early stages of IS. Metrics like CSF Influx and AQP4 polarization indicate impaired glymphatic function post-stroke, correlating with increased edema^5,6^. While these metrics start to recover around 7 days post-stroke and are linked to motor recovery at 3 months^7^, the dynamics of glymphatic function during stroke recovery and its impact on long-term cognitive outcomes, especially post-stroke cognitive impairment (PSCI), remain unclear.

Advances in neuroimaging permit non-invasive assessment of glymphatic system in stroke patients in clinical practice. To evaluate dynamics of glymphatic function during stroke recovery, we employed three non-invasive imaging markers for reflecting distinct aspects of the glymphatic system. First, choroid plexus (CP) volume is a hydrodynamic regulator propelling CSF-ISF exchange. Second, enlarged perivascular spaces (PVS), an established proxy for glymphatic stagnation, serve as structural indicators of impaired interstitial waste clearance, where severe PVS dilation correlates with glymphatic dysfunction^8^. Third, Diffusion Tensor Imaging Analysis along the Perivascular Space (DTI-ALPS) method, initially proposed by Taoka et al.^9^, quantifies the diffusion of water within PVS along deep medullary veins and has robust correlations with glymphatic clearance, as determined by dynamic contrast-enhanced imaging^10^. Emerging translational evidence highlights the clinical utility of these biomarkers in stroke populations. Jianming et al. have reported that the choroid plexus work as a site of damage in hemorrhagic and ischemic stroke^11^. PVS burden was also reported as a biomarker of neuropathological condition^12^. As for DTI-ALPS index, a recent clinical study has demonstrated that acute-phase DTI-ALPS reductions predict critical pathological cascades^7^. Importantly, preliminary longitudinal data suggest a potentially recovery of DTI-ALPS index during the subacute phase post-stroke. However, critical gaps persist regarding long-term glymphatic function: existing studies predominantly focus on acute-phase dysfunction, leaving the chronic-stage recovery patterns and their cognitive implications unexplored. Notably, no prior investigation has systematically examined whether glymphatic function, as captured by DTI-ALPS index, PVS or CP volume, modulates the risk of PSCI.

In this study, we evaluate the performance and change of glymphatic system, and its association with stroke outcome. We hypothesize that post-stroke glymphatic dysfunction exhibits time-dependent recovery patterns, and that this recovery correlates with stroke outcome. We aime to: (1) characterize the longitudinal evolution of DTI-ALPS indices, PVS burden, and CP volumes from subacute to chronic phases post-stroke; and (2) determine whether these biomarkers predict functional outcome and PSCI at 6-month follow-up.

## Materials and methods

### Participants

We enrolled two cohort population including non-stroke and stroke cohorts. Non-stroke cohorts were comprised by healthy controls (HC) and nonvascular cognitive impairment (n-VCI) patients.

#### Non-stroke cohorts

##### Healthy controls cohort

This cohort included 48 healthy controls who were selected from a study on 10-year dementia risk in young and middle-aged CSVD patients (DREAM-10, ClinicalTrials.gov: NCT06164262). Healthy controls were defined as individuals without subjective cognitive decline (MMSE scored higher than 26) or structural damage to the nervous system (CSVD burden score = 0) at the time of inclusion. The inclusion and exclusion criteria for FRESH-CSVD was shown in the supplementary file.

##### Non-vascular cognitive impairment (n-VCI) patients

This cohort included 47 n-VCI patients who were selected from a prospective observational study on fundus blood flow in patients with cognitive impairment (FRESH-CSVD, ClinicalTrials.gov: NCT06431711). According to the 2011 AHA/ASA scientific statement, vascular cognitive impairment (VCI) is defined as a syndrome characterized by clinical stroke or subclinical vascular brain injury, along with cognitive impairment affecting at least one cognitive domain. Cognitive impairment that does not meet the criteria for VCI is classified as n-VCI. The inclusion and exclusion criteria for FRESH-CSVD was shown in the supplementary file.

#### Stroke cohort

This cohort enrolled 51 patients with ischemic stroke who achieved successful reperfusion (modified thrombolysis in cerebral infarction < 2b) through thrombectomy at Zhejiang Provincial People’s Hospital between January 2018 and January 2024 (2017KY021). The inclusion criteria were: (i) a modified Rankin Scale (mRS) score of less than 2 prior to the stroke; (ii) unilateral anterior circulation ischemic stroke; (iii) no significant hemorrhagic transformation (parenchymal hemorrhage type according to the European Cooperative Acute Stroke Study classification). The exclusion criteria included: (i) patients with an unknown time of stroke onset; (ii) history of cerebral hemorrhage, subarachnoid hemorrhage, cranial trauma, psychiatric disorders, arteriovenous malformations, cerebral aneurysms, or brain tumors; (iii) a history of major neuropsychiatric disorders (e.g., Alzheimer’s disease, Parkinson’s disease, schizophrenia, and epilepsy); (iv) participation in drug clinical trials; and (v) incomplete imaging data or data affected by head motion artifacts.

All 51 patients followed a one-month rehabilitation program in our neurologic rehabilitation center consisting of intensive physiotherapy and occupational therapy 5 days a week. All patients received routine medications and rehabilitation training in the department, including comprehensive training of hemiplegic limbs, occupational therapy, physical factor therapy, acupuncture treatment, etc., using subjective, objective, assessment and plan (SOAP) mode to assess the patient’s function and develop treatment goals and reasonable treatment plans. Training frequency is 5 times a week, 40 min per time, for 1 month (4 weeks).

Of 51 patients, 41 underwent 1-month follow-up MRI scan and 6-month mRS and the Mini-Mental State Examination (MMSE) assessment. Post-stroke cognitive impairment (PSCI) refers to cognitive impairment that appears after a stroke event and persists until 6 months post-event. Cognitive impairment is defined according to the MMSE score as follows: when the total score of the MMSE is ≤ 20 for individuals with a low education level (≤ 6 years), and ≤ 24 for those with a high education level (> 6 years)^13,14^. All clinical information was extracted from the electronic database. Stroke features were evaluated and shown in the Supplementary file (See supplementary Methods).

### MRI acquisition

All participants underwent MRI scans using the same eight-channel phased array head coil on a 3.0 Tesla whole-body MRI scanner (Discovery MR750 3.0 T; GE Healthcare), followed the standardized protocol: 3DT1, T2-weighted, fluid-attenuated inversion recovery (FLAIR), diffusion weighted imaging, as well as DTI. For stroke cohort, 51 patients had an initial MRI scan before rehabilitation therapy (Time 1), and 41 had a follow-up MRI scan one month later (Time 2). The parameters of MRI sequences were shown in Supplementary Table I.

### Evaluation of glymphatic system

#### DTI-ALPS index

##### Processing and measurement method for obtaining the DTI-ALPS index

**Fig. 1:**
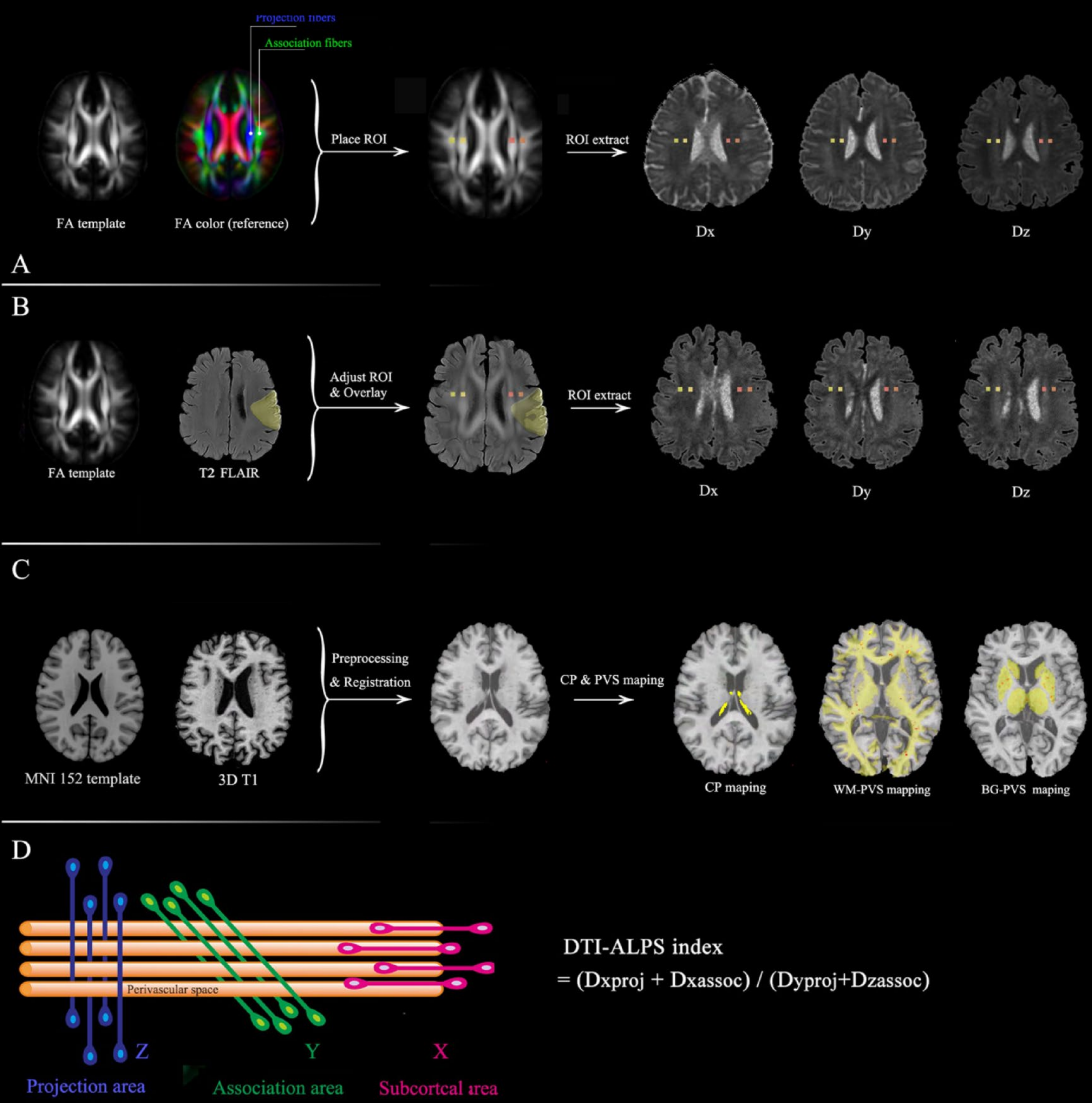
The assessment process for the glymphatic function by calculating the DTI-ALPS index in non-stroke cohorts (**A**) and stroke cohort (**B**), and CP and PVS volume (**C**). The goal for the placement of the ROIs (yellow and orange rectangles) is to place them on their bilateral projection fibers (blue area) and association fibers (green area), respectively (**D**) in calculating DTI-ALPS index. The relationship between the direction of the perivascular space (orange cylinder) and the direction of the fibers. ALPS, diffusion tensor image analysis along the perivascular space; FA, fractional anisotropy; FLAIR, fluid-attenuated inversion recovery; ROI, region of interest; MNI, Mapping-Montreal Neurological Institute; CP, choroid plexus; PVS, perivascular space; WM, white matter; BG, basal ganglia.

For preprocessing of brain MR-DTI images, FMRIB Software Library (FSL) software (version 6.0.5.3, https://fsl.fmrib.ox.ac.uk/fsl/fslwiki) was utilized for eddy currents, motion correction, skull stripping, and tensor fitting. The DTI-ALPS index was evaluated using the method described by Han et al.^15^, which allows for the calculation of the DTI-ALPS index without the need for susceptibility weighted imaging. The regions of interest (ROI) on DTI were evaluated using the location described by Han et al.^15^ The assessment process for the glymphatic function by calculating the DTI–ALPS index of non-stroke participants was shown in Fig. 1A. In pursuit of ROI placement satisfaction, we used T2 FLAIR images to adjust the ROIs slightly to avoid the influence of visibly damaged tissue (Fig. 1B). A Mapping-Montreal Neurological Institute (MNI) fractional anisotropy (FA) template (JHU-ICBM-FA-1 mm) (https://neurovault.org/images/1402/) was used for precise registration. Registration from the MNI space to the individual space was achieved through rigid registration followed by nonlinear registration using advanced normalization tools (ANTs)^16^ (https://stnava.github.io/ANTs/).

##### Calculation of the DTI-ALPS index

The DTI-ALPS index was proposed by Taoka et al.^9^. They described the relationship among projection fibers, association fibers and subcortical fibers (Fig. 1C). The DTI-ALPS index is calculated as the ratio between the mean diffusivity in the area of projection fibers (Dxproj) and association fibers (Dxassoc) on the x-axis and that of the projection fibers (Dyproj) on the y-axis and the association area (Dzassoc) on the z-axis:

$${\text{ALPS index}} = \frac{{{\text{mean}}\left( {{\text{Dxproj}},{\text{ Dxassoc}}} \right)}}{{{\text{ mean}}\left( {{\text{Dyproj}},{\text{ Dzassoc}}} \right)}}$$

For stroke patients, the DTI-ALPS index for both the infarct side and contralateral side were recorded separately. For HC and n-VCI cohorts, we calculated the DTI-ALPS values for the left and right sides first, and then recorded the average value of both sides to represent the DTI-ALPS index for each participant.

#### Quality control

Each individual ROI was visually inspected by two neurologists (with over 7 years of neuroimaging analysis experiences) who were blinded to the clinical data to confirm the accuracy of the location by ensuring that only blue voxels were included in the ROIs on the projected fibers and that only green voxels were included in the ROIs on the associated fibers.

#### PVS mapping

The PVS mapping was conducted following the methodology described by Sepehrband et al.^17^. Structural images, comprising 3D T1w and T2w images, underwent correction for gradient nonlinearity, readout, and bias field. Subsequently, these images were registered to the MNI 152 T1 template^18^ using FMRIB Software Library (FSL)^19^ version 5.0. A subsequent noise reduction process was implemented to target high-frequency spatial artifacts using a 1-voxel radius filtering patch^20^. The Frangi filter^21^, applied through the Quantitative Imaging Toolkit^22^, effectively estimated vesselness at different scales in order to enhance vessel detection in the 3D T1w images. The scale range was specifically established between 0.1 and 5 voxels to optimize vessel inclusion likelihood. A vesselness threshold of 0.0002 was applied to create the PVS mask. Each segmented PVS voxel was matched with corresponding T2-weighted (T2w) voxels to form the final PVS mask. A specific mask was developed in accordance with the anatomical boundaries of the basal ganglia (BG), delineated by the anterior insular tip and the posterior thalamic end. The standardized BG-PVS ratio was determined by normalizing against the Total Intracranial Volume (TIV). Similarly, the WM-PVS ratio was calculated after normalization, excluding cerebellar white matter.

#### CP mapping

In our study, we utilized 3D T1-weighted MRI sequences for the automated segmentation of the CP. After skull-stripping and intensity correction to ensure image consistency, the images were registered non-linearly to the MNI 152 T1-weighted template using the ANTs software package. The CP volume was calculated using a 3D U-Net deep learning model, as previously described by Eisma et al.^23^. CP labels were then transformed back to each subject’s original T1 space and validated by two expert neuroradiologists.

### Cerebral small vessel disease (CSVD) imaging marker measurements

Neuroimaging biomarkers of CSVD were defined according to the Standards for Reporting Vascular Changes on Neuroimaging 2 (STRIVE-2)^24^. The WMHs were rated using the Fazekas scale on axial brain FLAIR sequences, with a range of 0–3. Lacunes were evaluated on T1 and FLAIR images. Patients were divided into two groups: no lacunes and lacunes ≥ 1. EPVS were rated using a 4-point visual rating scale (0 = no EPVS, 1 = 10 EPVS, 2 = 11–20 EPVS, 3 = 21–40 EPVS, and 4 =  > 40 EPVS) validated for the basal ganglia and centrum semiovale regions^25^. We used the simplified visual Pasquier scale, which categorizes the severity of global cortical atrophy (GCA) as follows: 0- no atrophy; 1-mild atrophy; 2-moderate atrophy; and 3-severe atrophy^26^. The interobserver reliability kappa coefficients of the CSVD characteristics between two observers who were blinded to clinical data were as follows: WMH, 0.921; lacune, 0.951; EPVS, 0.861; and GCA, 0.825. The correlation between EPVS visual rating scale and bilateral BG-PVS volume were excellent (ρ = 0.180, *P* = 0.037).

### Statistical analysis

All analyses were performed using Empower (R) (www.empowerstats.com., X&Y solution, Inc., Boston, MA) and R (http://www.R-project.org). Interobserver reliability of all imaging features were assessed using the intraclass correlation coefficient. Numeric variables are expressed as the mean ± SD or median (IQR), while categorical variables are presented as frequencies (percentages). The Fisher’s exact test, the independent sample two-tailed t test and the Mann‒Whitney U test was used for different variables as appropriate. To account for multiple testing, two-sided *P* values were adjusted according to the method of Benjamin/Hochberg (B/H) to control the false discovery rate (FDR). An association was considered to be statistically significant, if its corresponding B/H-adjusted *P* value was below 0.05, corresponding to an FDR of 5%. Linear regression analysis was conducted to evaluate the relationship between the imaging markers of glymphatic system with stroke hemisphere and stroke prognosis. Multivariable linear regression analyses were performed after adjusting for potential confounders found in the univariate linear regression analysis (*P* < 0.05). In stroke cohort, a smoothing function was used to explore the relationship between the change of imaging markers of glymphatic system between Time 1 and Time 2 and the days since stroke onset (OTD).Statistical significance was set at 0.05 level (two-tailed).

## Results

### Study population and characteristics in two cohorts

A total of 146 participants were ultimately included in this analysis, consisting of 48 healthy controls (non-stroke cohorts), 47 patients with n-VCI (Non-Stroke cohorts), and 51 ischemic stroke patients (Stroke cohort). The average age was 63 (range: 12–94) years, and 82 participants (56.2%) were female. Among the participants, 45 (30.8%) had hypertension, 24 (16.4%) had diabetes, 6 (4.1%) had AF, and 31 (21.2%) had a history of stroke. The general clinical data of all participants is presented in Table 1**.**

**Table 1:** General clinical and imaging data of all participants (n = 146).

Characteristics	All participants, n = 146^1^	Non-Stroke cohorts		Stroke cohort n = 51^1^
		Healthy controls, n = 48^1^	n-VCI, n = 47^1^	
Female	82 (56.2)	35 (72.9)	31 (66.0)	16 (31.4)
Age, years,	63 (39–73)	42 (36–58)	71 (64–78)	64 (51–73)
Baseline MMSE	25 (15–30)	30 (27–30)	20 (18–25)	13 (0–26)
Stroke risk factors				
Previous stroke	31 (21.2)	0 (0.0)	0 (0.0)	31 (60.8)
Hypertension	45 (30.8)	7 (14.6)	5 (10.6)	33 (64.7)
DM	24 (16.4)	3 (6.2)	5 (10.6)	16 (31.4)
AF	6 (4.1)	0 (0.0)	0 (0.0)	6 (11.8)
Current smoking	24 (16.4)	3 (6.2)	4 (8.5)	17 (33.3)
Current drinking	18 (12.3)	2 (4.2)	3(6.4)	13 (25.5)
CSVD imaging markers				
Degree of WMH				
0	40 (27.4)	28 (58.3)	5 (10.6)	7 (13.7)
1	75 (51.4)	20 (41.7)	26 (55.3)	29 (56.9)
2	20 (13.7)	0 (0.0)	11 (23.4)	9 (17.6)
3	11 (7.5)	0 (0.0)	5 (10.6)	6 (11.8)
Degree of EPVS				
0	59 (40.4)	37 (77.1)	13 (27.7)	9 (17.6)
1	53 (36.3)	9 (18.8)	18 (38.3)	26 (51.0)
2	23 (15.8)	2 (4.2)	9 (19.1)	12 (23.5)
3	7 (4.8)	0 (0)	3 (6.4)	4 (7.8)
4	4 (2.7)	0 (0)	4 (8.5)	0 (0)
GCA score				
0	31 (21.2)	25 (52.1)	2 (4.3)	4 (7.8)
1	71 (48.6)	18 (37.5)	22 (46.8)	31 (60.8)
2	40 (27.4)	5 (10.4)	21 (44.7)	14 (27.5)
3	4 (2.7)	0 (0.0)	2 (4.3)	2 (3.9)
Lacune ≥ 1	43 (29.5)	2 (4.2)	7 (14.9)	34 (66.7)

n is the number of non-missing value.1 continuous variable, IQR; categorical variable, n (%). *IQR* interquartile range, *SD* standard deviation, *n-VCI* nonvascular cognitive impairment, *MMSE* Mini-Mental State Examination, *DM* diabetes mellitus, *AF* atrial fibrillation, *WMH* white matter hyperintensity, *EPVS* enlarged perivascular spaces.

### The comparisons among DTI-ALPS index, CP and PVS volume ratios in non-stroke and stroke cohorts

In non-stroke cohorts, compared with HC, n-VCI patients had lower mean values of DTI-ALPS index but higher CP volume ratio, while there was no difference in BG-PVS and WM-PVS volume ratio. In the stroke cohort at Time 1, compared with the non-infarct side, the infarct side had a lower DTI-ALPS index and a higher BG-PVS volume ratio. Compared with non-stroke cohorts, CP volume ratio of the infarct side was higher than that of the non-infarct side, and both the infarct and non-infarct sides had lower DTI-ALPS index in the stroke cohort, but only the infarct side of DTI-ALPS index had statistical significance (FDR *P* < 0.05). In the stroke cohort at Time 2, the non-infarct side displayed a slight improvement in DTI-ALPS index compared to Time 1, though not statistically significant. Conversely, there was a significant increase in DTI-ALPS of the infarct side. The CP and PVS volume ratios did not exhibit significant changes between the Time 1 and 2 assessments (Table 2).

**Table 2:** Description of the DTI-ALPS index levels in three cohorts.

	Non-stroke cohorts (n = 95)			Stroke cohort (n = 51)							
				Time 1 (n = 51)					Time 2 (n = 41)		
	Mean value	Healthy controls (n = 48)	n-VCI (n = 47)	Non-infarct side	FDR *P* value^#^	Infarct side	FDR* P* value^&^	Non-infarct side*	FDR *P* value*	Infarct side	FDR *P* value*
DTI-ALPS index	1.39 ± 0.24	1.45 ± 0.22	1.34 ± 0.25	1.32 ± 0.27	0.072	0.98 ± 0.30	< 0.001	1.33 ± 0.25	0.832	1.09 ± 0.22	0.046
CP volume ratio	0.15 ± 0.04	0.13 ± 0.03	0.16 ± 0.03	0.13 ± 0.03	0.142	0.14 ± 0.04	0.449	0.14 ± 0.03	0.296	0.14 ± 0.04	0.475
BG-PVS volume ratio	0.02 ± 0.01	0.02 ± 0.01	0.02 ± 0.01	0.01 ± 0.01	0.004	0.02 ± 0.02	0.789	0.02 ± 0.01	0.360	0.02 ± 0.01	0.502
WM-PVS volume ratio	0.10 ± 0.03	0.10 ± 0.02	0.10 ± 0.03	0.09 ± 0.03	0.072	0.09 ± 0.03	0.283	0.09 ± 0.04	0.593	0.09 ± 0.03	0.604

^&^Time 1 infarct side *vs.* non-stroke cohorts.

^#^Time 1 non-infarct side *vs.* non-stroke cohorts.

*Time 2 vs. Time 1.

*Mean value* the mean value of bilateral level of each imaging marker, *DTI-ALPS* diffusion tensor image analysis along the perivascular space, *n-VCI* nonvascular cognitive impairment, *CP* choroid plexus, *PVS* perivascular space, *BG* basal ganglia, *WM* white matter, *FDR* false discovery rate.

**Fig. 2:**
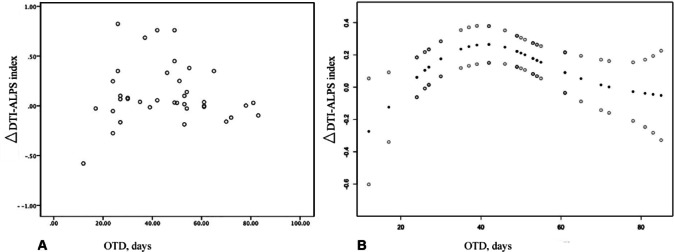
The relationship between the ΔDTI-ALPS index of infarct side and OTD was determined by smooth curve fitting. The scatter plot of the distribution of the ipsilateral ΔDTI-ALPS index and OTD (**A**) and the curve fitting line for the ΔDTI-ALPS index of infarct side and OTD (**B**) are shown. Adjusted variables: Age and number of lacunes. ΔDTI-ALPS index, changes in the DTI-ALPS index between Time 1 and Time 2 MRI scan; ODP, the days from stroke onset to the Time 1 MRI scan.

We further analyzed the difference between the DTI-ALPS index at Time 1 and Time 2 on the infarct side (ΔDTI-ALPS index of infarct side) and the correlation between the interval from onset to detection time (OTD) of patients. The linear regression analysis revealed that the OTD was not associated with the **Δ**DTI-ALPS index of infarct side (*P* > 0.05), but there was a nonlinear relationship between the **Δ**DTI-ALPS index of infarct side and the OTD (Fig. 2). When the OTD was less than 42 days, there was no significant association between the **Δ**DTI-ALPS index of infarct side and the OTD (β = 0.010, 95% CI = − 0.001–0.021; *P* = 0.095). However, when the OTD exceeded 42 days, the **Δ**DTI-ALPS index of infarct side significantly decreased with increasing OTD (β = − 0.004, 95% CI = − 0.008- -0.001,* P* = 0.024) after adjusting for age and lacunes (Table 3).

**Table 3:** Threshold effect analysis for the relationship between the ΔDTI-ALPS index of infarct side and OTD.

Models	**Δ**DTI-ALPS index of infarct side	*P* value	Adjusted β (95% CI)	*P* value
	Crude β (95% CI)			
Model I				
One line slope	− 0.001 (− 0.006, 0.004)	0.800	− 0.002 (− 0.005, 0.001)	0.219
Model II				
Turning point	42		42	
< 42 slope 1	0.029 (0.011, 0.047)	0.0032	0.010 (− 0.001, 0.021)	0.095
> 42 slope 2	− 0.009 (− 0.016, − 0.002)	0.0106	− 0.004 (− 0.008, − 0.001)	0.024
LRT test		0.001		0.016

Model I. Linear analysis; Model II, nonlinear analysis. LRT test, logarithmic likelihood ratio test. A *P* value < 0.05 indicated that Model II was significantly different from Model I, which indicated a nonlinear relationship. Crude: no adjustment; Adjusted: adjusted for age and number of lacunes. ΔDTI-ALPS index, changes in the DTI-ALPS index between Time 1 and Time 2 MRI scan; ODP, the days from stroke onset to the Time 1 MRI scan.

### Impact of ischemic stroke on DTI-ALPS index

**Fig. 3:**
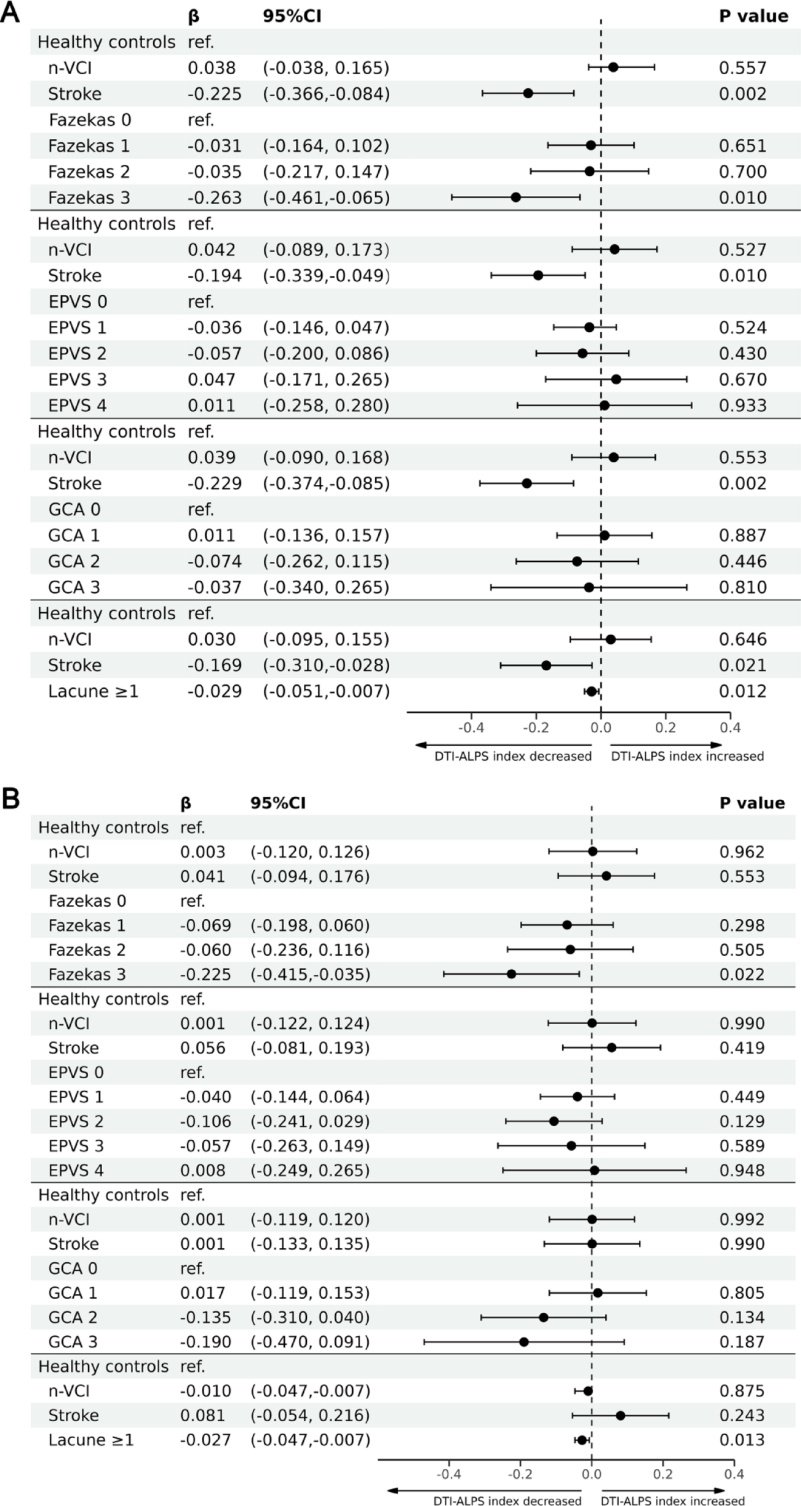
Linear regression analysis models for evaluating the association of stroke and each CSVD imaging marker with the DTI-ALPS index after adjusting for age, baseline MMSE score and hypertension status when combining the DTI-ALPS index of infarct side (**A**) or the contralateral side (**B**) with the DTI-ALPS index of non-stroke patients. DTI-ALPS, diffusion tensor image analysis along the perivascular space; n-VCI, nonvascular cognitive impairment; CSVD, cerebral small vessel disease; EPVS, enlarged perivascular space; 95% CI, 95% confidence interval.

When analyzing the Time 1 DTI-ALPS index of infarct side in stroke patients with the DTI-ALPS index of HC and n-VCI cohort population, we found that stroke was an independent factor associated with the DTI-ALPS index whenever combined with any of the CSVD imaging features (all *P* < 0.05). A Fazekas grade of 3 (compared to a Fazekas grade of 0) (*P* = 0.010) and lacunes (*P* = 0.012) were also independently associated with the DTI-ALPS index (Fig. 3A). When replacing the Time 1 DTI-ALPS index of infarct side with DTI-ALPS index of non-infarct side, we found no association between stroke and the DTI-ALPS index (all *P* > 0.05), while a Fazekas grade of 3 (compared to a Fazekas grade of 0) (*P* = 0.022) and lacunes (*P* = 0.013) were still independent factors influencing the DTI-ALPS index (Fig. 3B).

### Association between imaging markers of glymphatic system and stroke prognosis

In the stroke cohort at Time 1, the DTI-ALPS index of infarct side was both associated with Time 1 MMSE (r = − 0.507, *P* < 0.001) and NIHSS score (r = − 0.280, *P* = 0.046), and bilateral WM-PVS volume ratio were also associated with Time 1 MMSE score (infarct side: r = 0.435, *P* = 0.003; non-infarct side: r = 0.400, *P* = 0.007), while the remaining of glymphatic metrics did not exhibit significant associations with Time 1 MMSE, NIHSS score and infarct volume (all *P* > 0.05).

Of the 41 stroke patients with a Time 2 MRI scan, 26 (63.4%) had poor outcomes at 6 months. The univariate correlation analysis revealed that none of three glymphatic metrics at Time 1 and the **Δ**DTI-ALPS index of infarct side was associated with 6-months poor outcome (all *P* > 0.05). However, the Time 2 DTI-ALPS index of infarct side showed a significant correlation with 6-months poor outcome (ρ = − 0.467, *P* = 0.003) (Supplementary Table II). The binary logistic regression analysis demonstrated that the Time 2 DTI-ALPS index of infarct side (OR = 0.001, *P* = 0.018) was independently correlated with 6-months poor outcome. After adjusting for the Time 1 DTI-ALPS index of infarct side, the Time 2 DTI-ALPS index of infarct side remained independently correlated with 6-months poor outcome (OR = 0.001, *P* = 0.021) (Supplementary Table III). The sensitivity analysis found consistent results among patients with CSVD, revealing a stable association between the DTI-ALPS index of the infarcted side at Time 1 and Time 2, and 6-months poor outcomes (Supplementary Table IV). ROC curve analysis revealed that the Time 2 DTI-ALPS index of infarct side had good predictive value (AUC = 0.786, 95%CI = 0.624–0.947) for 6-month poor outcome after stroke, with a cutoff value of 1.105 (sensitivity: 0.85, specificity: 0.73) (Supplementary Fig. IA). DCA demonstrated that the Time 2DTI-ALPS of infarct side yielded a greater net benefit (Supplementary Fig. IB).

Regarding cognitive status, 26 patients (51%) at 6-months after stroke still performed as PSCI. univariate analysis showed that DTI-ALPS index of infarct side and bilateral WM-PVS volume ratio at both Time 1 and 2 were associated with 6-month MMSE score (all *P* < 0.05), while the rest imaging markers were not significant (all *P* > 0.05). The linear regression analysis revealed that the Time 1 DTI-ALPS index of infarct side and bilateral WM-PVS volume ratio were significantly associated with the 6-month MMSE score after adjusting for baseline NIHSS score and infarct volume (all FDR *P* < 0.05). Further regression analysis showed that, only DTI-ALPS index of the infarct side at both Time 1 and 2 were associated with PSCI (Table 4). The sensitivity analysis confirmed a stable association between the DTI-ALPS index of the infarcted side at Times 1 and 2 and 6-month PSCI in CSVD patients (Supplementary Table V).

**Table 4:** Imaging markers of glymphatic system that associated with 6-months MMSE and PSCI.

	Time 1			Time 2		
	B	95%CI	FDR* P*	B	95%CI	FDR* P*
6-months MMSE						
DTI-ALPS index of infarct side	14.091	2.841–25.341	0.031	8.799	− 8.063–25.660	0.522
WM-PVS volume ratio of infarct side	1.432	0.417–2.447	0.045	0.905	− 0.096–1.906	0.213
WM-PVS volume ratio of non-infarct side	1.564	0.415–2.714	0.03	0.987	0.127–1.848	0.155
6-months PSCI						
DTI-ALPS index of infarct side	0.001	0.001–0.122	0.045	0.005	0.001–0.849	0.215
WM-PVS volume ratio of infarct side	0.779	0.547–1.108	0.413	0.830	0.641–1.076	0.397
WM-PVS volume ratio of non-infarct side	0.794	0.524–1.204	0.463	0.813	0.644–1.026	0.135

Adjust for baseline NIHSS score and infarct volume.

*PSCI* post-stroke cognitive impairment, *CI* confidential interval, *DTI-ALPS* diffusion tensor image analysis along the perivascular space, *PVS* perivascular space, *WM* white matter, *FDR* false discovery rate.

## Discussion

In this study, we investigated differences in glymphatic function, as measured by the DTI-ALPS index, along with CP and PVS volume ratios in a heterogeneous cohort comprising healthy controls, patients with n-VCI, and ischemic stroke patients. Our results showed that the infarct side in stroke patients exhibited significantly lower DTI-ALPS index values compared to both the non-infarct side and non-stroke cohorts. These findings indicate that ischemic injury may lead to an impaired glymphatic clearance mechanism. The Time 1 decreased DTI-ALPS index on the infarct side and its association with 6 months MMSE and the PSCI further underscore the clinical relevance of measuring glymphatic dysfunction in the context of acute cerebrovascular events.

In comparison analyses, we found a variation in DTI-ALPS index levels among different populations, with no statistically significant differences in PVS and CP volume ratios among cohorts. Bilateral reductions in the DTI-ALPS index were observed in the stroke cohort compared to the non-stroke cohort, with a more significant decline on the infarct side. We further investigated factors associated with the bilateral reduced DTI-ALPS index and found that it was correlated with the severity of WMH. The finding suggests that bilateral reduction in the DTI-ALPS index is likely linked to pre-existing CSVD severity, with a reduction on the infarct side attributed to the combined impact of stroke and CSVD.

Previous studies have documented impairments in CSF secretion, dynamic disturbances, and increased CP volume in populations with neurodegenerative diseases^27,28^. Following a stroke, compared to HC, CP volume significantly increases and remains elevated for up to 12 months without reversal^29^. In this study, although no significant difference in CP was observed between stroke and non-stroke populations, the CP volume ratio in stroke patients was slightly lower than that in n-VCI individuals. Compared to HC, stroke patients exhibited a mild larger CP volume on the infarct side, and this volume did not change over time. This finding aligns with previous research, but our study did not detect a statistically significant larger CP volume in the stroke cohort compared to the HC. This discrepancy may be attributed to the more refined methodology employed in this study and the limited sample size. Future research could further validate these findings by expanding the sample size.

Our results also revealed that the decline in the DTI-ALPS index on the infarct side showed a significant recovery over time, indicating that the DTI-ALPS index retains the potential for recovery even in the chronic stage of stroke. The recovery process is nonlinear, displaying an inverse U-shaped relationship between the degree of DTI-ALPS index recovery and the time since stroke onset (ODT). Notably, around the 42-day mark, there may be a decline in glymphatic function recovery ability. However, the 42-day threshold identified for glymphatic recovery is statistically derived and lacks physiological validation. Future research will evaluate the time points of glymphatic system recovery in larger clinical studies and validating these findings with physiological experimental results.

Notably, though the Time 1 DTI-ALPS index of the infarct side did not predict 6-month poor outcome, the Time 2 index did. The mechanism underlying this phenomenon is as follows: (1) The repairment of glymphatic function is time-dependent. Rehabilitation interventions, such as increased physical activity and neurostimulation, may enhance CSF flow through arterial vasomotion^30^, thereby restoring perivascular flow. Improved DTI-ALPS index at Time 2 may indicate a restored waste clearance capacity, potentially reducing long-term neuroinflammation and secondary neurodegeneration; (2) Resolution of acute phase confounders are essential. Early post-stroke fluctuations in DTI-ALPS may be influenced by factors such as edema, microbleeds, or blood–brain barrier disruption. By Time 2, these transient disturbances resolve, revealing persistent glymphatic impairment. This stabilized metric may better predict irreversible drainage failure, which is associated with long-term outcomes.

Our study revealed that the Time 1 DTI-ALPS index of the infarct side exhibited predictive value for both MMSE score and PSCI at 6 months. The mechanisms behind these associations may involve: (1) Post-stroke loss of blood–brain barrier integrity^10^, which impacts the PVS structure and glymphatic system efficiency, leading to harmful substance accumulation (e.g., lactate, tau, Aβ) and dementia progression^31,32^. (2) Ischemic stroke reducing CSF circulation due to decreased arterial pulsation^33^, impairing glymphatic waste clearance and exacerbating Aβ accumulation, thus accelerating dementia development^34^. In clinical practice, DTI-ALPS index could be instrumental in stratifying patients based on their risk of developing cognitive decline and monitoring the effectiveness of rehabilitation interventions. In hospitals, adding a quick DTI scan (< 5 min) to standard stroke MRI could help doctors spot patients needing early cognitive therapy or experimental drugs that boost glymphatic clearance. Over time, repeating DTI scans during follow-ups might show if treatments are working at a biological level, beyond just testing memory. However, more studies across diverse patient groups are needed to confirm the best DTI-ALPS index cutoff values. Addressing these through standardized scanning protocols and large trials will be key to making DTI-ALPS index a routine tool for stroke rehabilitation. Furthermore, we found that WM-PVS volume ratio of the infarct side at Time 1 associated with the 6-month MMSE, but it failed to show a correlation with PSCI. In a retrospective study of 430 participants, Francesco Arba et al. identified a significant independent association between enlarged PVS in the basal ganglia and cognitive impairment one year post-stroke, even after adjusting for clinical factors and both clinical and imaging variables^35^. Additionally, Tu et al. demonstrated a significant link between early-onset cognitive decline (within one month post-stroke) and BG-EPVS^36^. However, there is currently a lack of reports on the association between WM-PVS and cognitive impairment in ischemic stroke patients. Our study identified WM-PVS, rather than BG-PVS, as the marker associated with 6-month MMSE. This discrepancy may stem from inadequate quantitative assessment of PVS and the absence of evaluation of WM-PVS severity in prior stroke studies, highlighting the need for further investigation.

This study has several limitations. First, it is a retrospective, single-center analysis with a small sample size. Second, the exclusion of severe stroke patients who were ineligible for MRI may bias the population by omitting those with severe glymphatic dysfunction (e.g., due to prolonged ischemia-induced irreversible AQP4 depolarization), potentially skewing our findings toward milder cases where glymphatic plasticity might persist. Data from a TBI study^37^ suggest injury severity dictates AQP4 recovery (for example, mild TBI shows normalization within 14–28 days whereas moderate TBI exhibits delayed or incomplete recovery), implying potential irreversibility in severe stroke. Future studies should recruit NIHSS-stratified cohorts and conduct sensitivity analyses to test the generalizability of the results. Third, the lack of age-matched non-stroke controls limits specificity. Incorporating such controls in future work could disentangle stroke-specific glymphatic effects from aging or comorbidity confounders. Furthermore, the utility of DTI-ALPS index should be interpreted with caution. While preclinical investigations have shown associations between DTI-ALPS and tracer clearance, the specificity of DTI-ALPS as a biomarker of PSCI remains to be validated (such as through joint validation with standard techniques like intrathecal gadolinium contrast imaging). As highlighted by Ringstad^38^, some studies have pointed out that CSF-ISF exchange in deep white matter may only account for a minor part of the glymphatic circulation, suggesting that the physiological significance of the DTI-ALPS index may vary regionally. Several limitations of the technique should be noted: (1) DTI-ALPS primarily assesses local perivenous water dynamics rather than comprehensive glymphatic function, potentially missing complex system interactions. (2) There is insufficient validation of preclinical findings in human pathophysiology, especially in stroke patients, necessitating cautious interpretation in clinical settings. (3) Post-stroke structural abnormalities can complicate DTI-ALPS index interpretation, introducing variability unrelated to glymphatic function. To address this issue, we have incorporated the quantification of CP and PVS burden into our study, which to some extent compensates for the limitations in assessing glymphatic function due to methodological limitation. Despite methodological debates ^38^, the predictive efficacy of DTI-ALPS index underscores the presence of clinically actionable perivascular recovery phenotypes that are yet to be fully characterized. Future research endeavors should combine ALPS metrics with emerging techniques like diffusion-prepared arterial spin labeling (DP-ASL) and computational models of CSF-ISF pressure gradients. Lastly, although we have identified the non-linear pattern of glymphatic recovery through statistical models, further biological mechanism research is needed to validate this phenomenon. Future studies should delve into the underlying mechanisms of the 42-day threshold to better understand the dynamic process of glymphatic function recovery.

## Conclusion

Compared to non-stroke populations, there is a significant decline in glymphatic function after stroke, primarily manifested by a marked decrease in the DTI-ALPS index on the infarct side, which gradually recovers over time. Pre-rehabilitation DTI-ALPS index cannot be used to predict functional outcomes, but it can predict MMSE and PSCI at 6 months, supporting its potential future application in the early screening of cognitive impairment after ischemic stroke.

## Supplementary Information

Supplementary Information.

## Data Availability

The datasets generated during the present study are available from the corresponding author upon reasonable request. Considerations will be made based on the review of reasons for requesting the data and the procedures for ensuring data privacy.
