# Supplementary material for: Glymphatic dysfunction as a biomarker for post-stroke cognitive impairment
Source: Sci Rep. 2025 Jun 3;15:19382. doi: 10.1038/s41598-025-04054-9 (PMC12134357; doi:10.1038/s41598-025-04054-9)
Supplement: Supplementary file 1 — Supplementary Information. [file 41598_2025_4054_MOESM1_ESM.docx]

**Supplementary files**

**Supplementary Methods**

**The inclusion and exclusion criteria for FRESH-CSVD and DREAM-10 study**

**FRESH-CSVD study (ClinicalTrials.gov: NCT06431711)**

Inclusion Criteria:

1.Older than 30 years old.

2.Patients who underwent Multimodal MRI with any CSVD imaging marker.

3.Subjects who have signed informed consent.

Exclusion Criteria:

1. Patient who was unable to cooperate with examinations.

2. There are known diseases that may cause or worsen CSVD (brain injury, Down syndrome, Alzheimer's disease, Parkinson's disease, etc.).

3. There are known eye diseases or severe underlying fundus lesions that may impact fundus assessment.

4. Suffering from serious systemic diseases, such as heart, liver, kidney diseases or major mental illnesses.

5. Contraindications for imaging examinations.

**DREAM-10 study (ClinicalTrials.gov: NCT06164262)**

Inclusion Criteria:

1. Patients with any of the CSVD-related MRI imaging markers, including recent small subcortical infarcts, lacunes, white matter hyperintensities, perivascular spaces, microbleeds, and superficial cortical siderosis.

2. Patients aged from 30 to 60 years.

3. Sign informed me of consent.

Exclusion Criteria:

1. Unable to cooperate with inspectors.

2. Known dementia.

3. Other cognitive diseases (such as Alzheimer's disease, Parkinson's disease, or thyroid disease).

4. Serious systemic illness, such as heart, liver, kidney disease or major mental illness.

5. Contraindications for imaging examination

**Stroke features**

Pretreatment brain CTA was used to evaluate collateral status and clot burden using the modified Tan’s scale^1^ and a 10-point scoring system (clot burden score, CBS) ^2^, respectively. Brain edema and infarct volume were assessed using 3-5 days brain non-contrast CT or MRI after stroke. Brain edema was independently assessed by two stroke neurologists using a 7-point scale to grade hemispheric swelling ^3^. Brain edema was defined as the scale score exceeding one point. The infarct volume was acquired by manual segmentation on non-contrast CT images or b1000 diffusion-weighted MRIs using commercial software (MIStar; Apollo Medical Imaging Technology, Melbourne, Australia) by a single neuroradiologist. Stroke etiology was assessed based on Trial of Org 10 172 in Acute Stroke Treatment (TOAST) classifications ^4^.

**References**

1. Yeo LLL, Paliwal P, Teoh HL, et al. Assessment of intracranial collaterals on CT angiography in anterior circulation acute ischemic stroke. *AJNR Am J Neuroradiol*. 2015;36(2):289-294. doi:10.3174/ajnr.A4117

2. L L, O N, G T, et al. Clot burden score on admission T2*-MRI predicts recanalization in acute stroke. *Stroke*. 2013;44(7). doi:10.1161/STROKEAHA.113.001026

3. Wardlaw JM, Sellar R. A simple practical classification of cerebral infarcts on CT and its interobserver reliability. *AJNR Am J Neuroradiol*. 1994;15(10):1933-1939.

4. Adams HP, Bendixen BH, Kappelle LJ, et al. Classification of subtype of acute ischemic stroke. Definitions for use in a multicenter clinical trial. TOAST. Trial of Org 10172 in Acute Stroke Treatment. *Stroke*. 1993;24(1):35-41. doi:10.1161/01.str.24.1.35

**Supplementary Table I. The detailed MRI scan parameters in the present study**

| Contrast | Orientation | Parameters |
| --- | --- | --- |
| T1w MPRAGE | 3D sagittal | TR=6.7ms, TE=2.9ms, TI=450ms, Flip angle=12°, Slice spacing=0mm, Slice thickness=1mm, Matrix size =256×256, FOV =256×256 mm^2^, 192 vectorial sections with 1×1×1mm^3^ voxels. |
| T2Flair | 2D axial | Fast spin‒echo sequence: TR=7500ms, TE=152ms, TI =2,100ms, Flip angle=90°, Slice spacing = 0mm, Slice thickness=4mm, Matrix size=320×256, FOV=24×24cm^2^. |
| T2W | 2D axial | TR=9,823ms, TE=101ms, Slice thickness=5mm, Slice spacing=1.5mm, FOV= 220×220mm^2^. |
| DWI | 2D axial | TR=4,000ms, TE=79ms, Flip angle=90°, Slice thickness=4mm, Matrix size=256×256, FOV=24×24cm^2^, b-value=1,000 s/mm2. |
| DTI | 2D axial | Spin-echo-planar imaging: TR=8,612ms, TE=63.8ms, Matrix size=256×256, Slice spacing=0mm, FOV=192×192 mm2, Maximum b-value=1000s/mm2, 30 non-collinear directions, 1vol was acquired without diffusion weighting (b-value = 0 s/mm2). |

T1w MPRAGE, T1-weighted magnetization prepared rapid acquisition gradient-echo; T2w, T2-weighted; FLAIR, fluid-attenuated inversion recovery; DWI, diffusion weighted imaging; DTI, diffusion tensor imaging; TR, time repetition; TE; time echo TI, inversion time.

**Supplementary Table II.** Factors associated with 6-month functional outcome

|  | Good outcome | Poor outcome | *P* value | OR | 95% CI | Adjusted *P* value |
| --- | --- | --- | --- | --- | --- | --- |
|  | (n=18) | (n=33) |  |  |  |  |
| Age, years, mean ± SD | 62.5 (53.7-72) | 65 (50.5-74) | 0.928 |  |  |  |
| Female, n (%) | 5 (27.8) | 11 (33.3) | 0.683 |  |  |  |
| Baseline NIHSS, median (IQR) | 6 (3.5-12.5) | 14 (11-17) | 0.001 | 1.287 | 0.991-1.672 | 0.059 |
| Baseline MMSE, median (IQR) | 25 (9-27) | 12 (0-22) | 0.034 | 1.025 | 0.938-1.119 | 0.587 |
| Stroke risk factors, n (%) |  |  |  |  |  |  |
| Previous stroke | 9 (50.0) | 22 (66.7) | 0.244 |  |  |  |
| Hypertension | 12 (66.7) | 21 (63.6) | 0.829 |  |  |  |
| DM | 6 (33.3) | 10 (30.3) | 0.824 |  |  |  |
| AF | 1 (5.6) | 5 (15.2) | 0.309 |  |  |  |
| Medicine, n (%) |  |  |  |  |  |  |
| Anti-hypertension drug | 13 (72.2) | 23 (69.7) | 0.850 |  |  |  |
| Anti-DM | 6 (33.3) | 10 (30.3) | 0.824 |  |  |  |
| Antiplatelet/anticoagulation | 11 (61.1) | 24 (72.7) | 0.393 |  |  |  |
| Statin | 11 (61.1) | 21 (63.6) | 0.859 |  |  |  |
| Imaging features of stroke |  |  |  |  |  |  |
| Poor collaterals, n (%) | 6 (33.3) | 20 (60.6) | 0.063 | 0.556 | 0.078-3.946 | 0.557 |
| CBS ≥6, n (%) | 16 (88.9) | 25 (75.8) | 0.259 |  |  |  |
| Brain edema, n (%) | 6 (33.3) | 15 (45.5) | 0.401 |  |  |  |
| Infarct volume, ml, mean ± SD | 21.0±25.0 | 29.5±30.8 | 0.318 |  |  |  |
| Stroke etiology, n (%) |  |  | 0.527 |  |  |  |
| LAA | 12 (66.7) | 20 (60.6) |  |  |  |  |
| CE | 0 (0) | 7 (21.2) |  |  |  |  |
| SAO | 3 (16.7) | 5 (15.2) |  |  |  |  |
| OE | 3 (16.7) | 0 (0) |  |  |  |  |
| UE | 0 (0) | 1(3.0) |  |  |  |  |
| SVD-related imaging markers, n (%) |  |  |  |  |  |  |
| Degree of WMH |  |  | 0.423 |  |  |  |
| 0 | 4 (22.2) | 3 (9.1) |  |  |  |  |
| 1 | 8 (44.4) | 21 (63.6) |  |  |  |  |
| 2 | 3 (16.7) | 6 (18.2) |  |  |  |  |
| 3 | 3 (16.7) | 3 (9.1) |  |  |  |  |
| Degree of EPVS |  |  | 0.471 |  |  |  |
| 0 | 3 (16.7) | 6 (18.2) |  |  |  |  |
| 1 | 10 (55.6) | 16 (48.5) |  |  |  |  |
| 2 | 5 (27.8) | 7 (21.2) |  |  |  |  |
| 3 | 0 (0.0) | 4 (12.1) |  |  |  |  |
| 4 | 0 (0.0) | 0 (0.0) |  |  |  |  |
| GCA score |  |  | 0.541 |  |  |  |
| 0 | 1 (5.6) | 3 (9.1) |  |  |  |  |
| 1 | 13 (72.2) | 18 (54.5) |  |  |  |  |
| 2 | 4 (22.2) | 10 (30.3) |  |  |  |  |
| 3 | 0 (0) | 2 (6.1) |  |  |  |  |
| Numbers of lacunes, median (IQR) | 1 (0-3) | 1 (0-3) | 0.708 |  |  |  |
| Lacune≥1 | 10 (55.6) | 24 (72.7) | 0.214 |  |  |  |
| Time 1 DTI-ALPS index of infarct side, mean ± SD | 1.1 ± 0.3 | 0.9 ± 0.3 | 0.179 |  |  |  |
| Time 1 DTI-ALPS index of contralateral side, mean ± SD | 1.3 ± 0.2 | 1.3 ± 0.3 | 0.839 |  |  |  |
| Time 2 DTI-ALPS index of infarct side, mean ± SD^a^ | 1.2 ± 0.2 | 1.0 ± 0.2 | 0.002 |  |  |  |
| Time 2 DTI-ALPS index of contralateral side, mean ± SD^a^ | 1.4 ± 0.2 | 1.3 ± 0.3 | 0.613 | 0.001 | 0.001-0.301 | 0.018 |
| ODT, days, mean ± SD | 37.8 ± 19.4 | 44.7 ± 20.3 | 0.249 |  |  |  |
| **Δ**DTI-ALPS index, mean ± SD^a^ | 0.1 ± 0.3 | 0.1 ± 0.3 | 0.722 |  |  |  |

a, n=41

NIHSS: National Institute of Health Stroke Scale; MMSE, Mini-Mental State Examination; DM, diabetes mellitus; AF, atrial fibrillation; WMH, white matter hyperintensity; EPVS, enlarged perivascular spaces; DTI-ALPS, diffusion tensor image analysis along the perivascular space; ODT, days from stroke onset to Time 1 MRI scan; **Δ**DTI-ALPS index, changes in DTI-ALPS index from Time 1 to Time 2 MRI scan; IQR, interquartile range.

**Supplementary Table III.** Multivariable Logistic regression analysis for factors associated with 6-months poor outcome after stroke

| Variables | OR | 95%CI | | *P* value |
| --- | --- | --- | --- | --- |
|  |  | Upper | Lower |  |
| Model I |  |  |  |  |
| Baseline NIHSS | 1.85 | 1.022 | 1.374 | 0.025 |
| Baseline MMSE | 0.992 | 0.919 | 1.071 | 0.842 |
| Poor collateral | 2.100 | 0.460 | 9.593 | 0.338 |
| Time 1 DTI-ALPS index of infarct side | 0.829 | 0.060 | 11.357 | 0.888 |
| Model II |  |  |  |  |
| Baseline NIHSS | 1.287 | 0.991 | 1.672 | 0.059 |
| Baseline MMSE | 1.025 | 0.938 | 1.119 | 0.587 |
| Poor collateral | 0.556 | 0.078 | 3.946 | 0.557 |
| Time 2 DTI-ALPS index of infarct side | 0.001 | 0.001 | 0.190 | 0.014 |
| Model III |  |  |  |  |
| Baseline NIHSS | 1.281 | 0.986 | 1.664 | 0.064 |
| Baseline MMSE | 1.029 | 0.937 | 1.131 | 0.546 |
| Poor collateral | 0.562 | 0.080 | 3.974 | 0.564 |
| Time 1 DTI-ALPS index of infarct side | 0.596 | 0.014 | 24.908 | 0.786 |
| Time 2 DTI-ALPS index of infarct side | 0.001 | 0.000 | 0.309 | 0.021 |

NIHSS: National Institute of Health Stroke Scale; MMSE, Mini-Mental State Examination; DTI-ALPS, diffusion tensor image analysis along the perivascular space; OR, odds ratio; 95%CI, 95% confidence interval.

Supplementary Table IV. The sensitivity analysis for the association between DTI-ALPS index of infarct side with 6-months poor outcome

|  | CSVD markers | Stratification | n | B | 95%CI | *P* value |
| --- | --- | --- | --- | --- | --- | --- |
| Time 1 DTI-ALPS index of infarct side | WMH | No | 7 | - | - | - |
|  |  | Yes | 44 | 0.208 | 0.010-4.347 | 0.311 |
|  | Brain Atrophy | No | 4 | - | - | - |
|  |  | Yes | 47 | 0.953 | 0.058-15.537 | 0.973 |
|  | EPVS | No | 9 | - | - | - |
|  |  | Yes | 42 | 0.903 | 0.037-22.086 | 0.950 |
|  | Lacune | No | 17 | 9.383 | 0.025-35.606 | 0.459 |
|  |  | Yes | 34 | 0.294 | 0.011-7.698 | 0.463 |
| Time 2 DTI-ALPS index of infarct side | WMH | No | 4 | - | - | - |
|  |  | Yes | 37 | 0.001 | 0.001-0.380 | 0.025 |
|  | Brain Atrophy | No | 3 | - | - | - |
|  |  | Yes | 39 | 0.001 | 0.001-0.278 | 0.019 |
|  | EPVS | No | 6 | - | - | - |
|  |  | Yes | 35 | 0.001 | 0.001-0.827 | 0.045 |
|  | Lacune | No | 11 | 0.001 | 0.001-51.758 | 0.281 |
|  |  | Yes | 31 | 0.001 | 0.001-0.388 | 0.026 |

DTI-ALPS, diffusion tensor image analysis along the perivascular space; CSVD, cerebral small vessel disease; WMH, white matter hyperintensity; EPVS, enlarged perivascular space.

Supplementary Table V. The sensitivity analysis for the association between DTI-ALPS index the infarct side with 6-months PSCI.

| PSCI | CSVD markers | Stratification | n | B | 95%CI | *P* value |
| --- | --- | --- | --- | --- | --- | --- |
| Time 1 DTI-ALPS index of infarct side | WMH | No | 7 | - | - | - |
|  |  | Yes | 44 | 0.001 | 0.001-0.393 | 0.025 |
|  | Brain Atrophy | No | 4 | - | - | - |
|  |  | Yes | 47 | 0.001 | 0.001-0.280 | 0.023 |
|  | EPVS | No | 9 | - | - | - |
|  |  | Yes | 42 | 0.001 | 0.001-0.337 | 0.027 |
|  | Lacune | No | 17 | - | - | - |
|  |  | Yes | 34 | 0.001 | 0.001-0.370 | 0.024 |
| Time 2 DTI-ALPS index of infarct side | WMH | No | 4 | - | - | - |
|  |  | Yes | 37 | 0.017 | 0.001-3.392 | 0.130 |
|  | Brain Atrophy | No | 3 | - | - | - |
|  |  | Yes | 39 | 0.007 | 0.001-1.508 | 0.070 |
|  | EPVS | No | 6 | - | - | - |
|  |  | Yes | 35 | 0.051 | 0.001-69.276 | 0.0419 |
|  | Lacune | No | 11 | - | - | - |
|  |  | Yes | 31 | 0.028 | 0.001-4.601 | 0.170 |

DTI-ALPS, diffusion tensor image analysis along the perivascular space; CSVD, cerebral small vessel disease; WMH, white matter hyperintensity; EPVS, enlarged perivascular space.


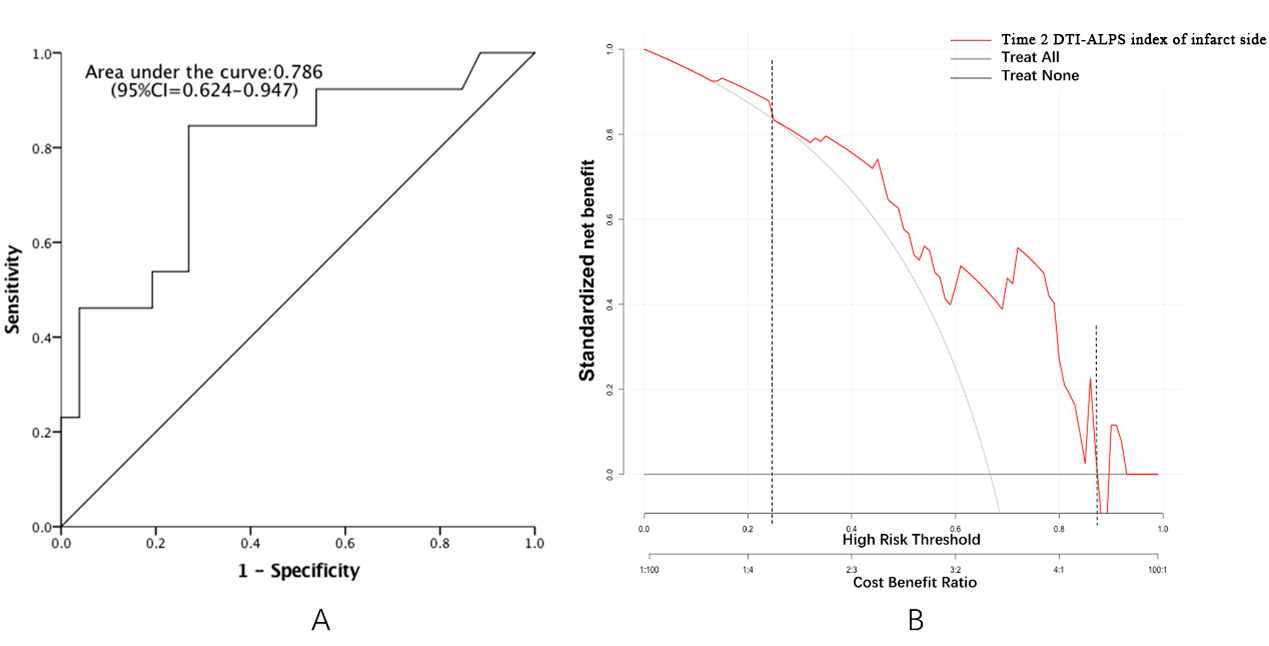


**Supplementary Fig. I.** The predictive value of Time 2 DTI-ALPS index of infarct side for poor outcomes through ROC analysis (A) and decision curve analysis (DCA) (B). ROC curve analysis revealed that the Time 2 DTI-ALPS index of infarct side had good predictive value (AUC=0.786, 95%CI=0.624-0.947) for 6-month poor outcome after stroke, with a cutoff value of 1.105 (sensitivity: 0.85, specificity: 0.73) (A). DCA demonstrated that the Time 2 DTI-ALPS of infarct side yielded a greater net benefit than did the treat-none or treat-all strategies when the threshold probability ranged from 25% to 87% (B).

DTI-ALPS, diffusion tensor image analysis along the perivascular space.
